# Transforming higher education: Embracing gender diversity for an inclusive future

**DOI:** 10.4102/safp.v68i1.6232

**Published:** 2026-01-05

**Authors:** Indiran Govender, Ramprakash Kaswa, Arun Nair

**Affiliations:** 1 Department of Family Medicine and Primary Health Care, Walter Sisulu University, Mthatha, South Africa; 2 Charles Sturt University, Australia; 3 Department of Family Medicine and Primary Health Care, Walter Sisulu University, Mthatha Regional Hospital, Mthatha, South Africa; 4 Department of Family Medicine, University of the Free State, Kimberley, South Africa; 5 Department of Family Medicine, Robert Mangaliso Sobukwe Hospital, Kimberley, South Africa

South Africa’s *Constitution (Act No. 108 of 1996)* affirms everyone’s right to live free from discrimination, which is based on sexual orientation and gender identity.^1^ Yet, transgender, non-binary, and other gender-diverse (TGD) individuals continue to face systemic barriers in higher education and health care.^2,3^ At the time of writing this editorial, South Africa commemorates Women’s Month, offering an opportunity to reflect on the evolving landscape of gender inclusivity within higher education. While progress has been made in advancing women’s rights and representation, inclusive practices and policies must also embrace the full spectrum of gender identities.^4^ Universities and training institutions must create inclusive environments and lead reforms to support TGD individuals, ensuring an equitable, affirming workforce.^5^ In the discipline of family medicine and primary care, embracing gender diversity is both a human rights issue and a professional obligation.^4^ This includes training academic and clinical staff to deliver respectful, evidence-based care, requiring changes in curricula, culture, and policies in alignment with gender diversity.^6^

Transgender, non-binary, and other gender-diverse individuals continue to be poorly understood within the wider socio-cultural context, leading to stigma and discrimination based on gender identity. Gender identity, sexual orientation, romantic preferences, and relationship choices are often confused and conflated, resulting in misconceptions and social stigma. These aspects are not directly related to biological sex.^4,5^ Transgender, non-binary, and other gender-diverse individuals may experience gender incongruence – a term used in ICD-11 to describe a persistent mismatch between a person’s gender identity and their sex at birth. When this incongruence causes significant distress, it may be diagnosed as gender dysphoria (DSM-5).^7^ It is estimated that around 1% of the general population experiences gender dysphoria. However, not all TGD people experience this condition, and labelling their identities as ‘gender dysphoria’ can worsen mental health issues. Many members of the TGD community have faced oppression, marginalisation, and transphobia. Discrimination rooted in transphobia greatly contributes to higher rates of social problems and mental health difficulties, including suicidal thoughts.^8,9^

In educational contexts, TGD students often face administrative barriers, social exclusion, and mental health issues. Misgendering, whether accidental or deliberate, undermines their dignity and well-being.^2,4^ However, with appropriate conditions, higher education can be a transformative experience for transgender individuals, offering opportunities for personal growth, academic achievement, and community involvement. Benefits include greater self-awareness, improved interpersonal relationships, and the chance for positive societal influence. Higher education institutions can foster inclusivity by establishing a welcoming campus atmosphere. They should implement inclusive policies that endorse students’ preferred names and pronouns, protect privacy, and guarantee access to gender-affirming facilities and support services.^3^

South African universities are beginning to respond. Initiatives have been taken that demonstrate efforts to move beyond the gender binary and tackle transphobia in higher learning environments.^2,10,11^ Several resources offer valuable guidance for institutions seeking to implement inclusive practices.

Key policy components include:

- Inclusivity and non-discrimination: Ensuring equal access to education and services for all students
- Preferred names and pronouns: Respecting affirmed identities in all communications and records
- Support services: Providing mental health and gender-affirming care tailored to TGD needs
- Training and awareness: Equipping staff and students with the knowledge to engage respectfully and professionally
- Legal compliance: Aligning with anti-discrimination legislation and ethical standards.

Transformation of curricula and institutions will lead current and future healthcare providers to recognise and affirm gender diversity without bias. This involves understanding the complexities of gender identity, avoiding assumptions, and creating safe spaces for disclosure. As highlighted in recent South African and international literature, inclusive care training reduces health disparities and enhances outcomes for sexual and gender minorities. Several global and local organisations advocate for gender-sensitive care and the integration of sexual health education into healthcare training curricula.^5,8,12^

The transformation of higher education and healthcare must be rooted in equity, diversity, and inclusion. This involves prioritising the voices and experiences of TGD students, educators, and patients. It also requires reassessing the significance of professionalism and ethics within gender-diverse teams and communities.^4,11^ Furthermore, it entails committing to lifelong learning and institutional accountability. Family physicians and primary care providers are often the first point of contact for healthcare needs. As educators, clinicians, and leaders in family medicine, we have a duty to create and foster environments where all individuals, regardless of gender identity, feel affirmed, respected, and empowered.
